# Molecular detection and *pyr*G sequence analysis of *Avibacterium paragallinarum* using clinical samples of infraorbital exudates from layer chickens with infectious coryza symptoms in Indonesia

**DOI:** 10.14202/vetworld.2023.1655-1660

**Published:** 2023-08-17

**Authors:** Fadhli Nanda Putra, A. E. T. H. Wahyuni, Bambang Sutrisno

**Affiliations:** 1. Department of Microbiology, Faculty of Veterinary Medicine, Universitas Gadjah Mada, Yogyakarta 55281, Indonesia; 2 Department of Pathology, Faculty of Veterinary Medicine, Universitas Gadjah Mada, Yogyakarta 55281, Indonesia

**Keywords:** *Avibacterium paragallinarum*, bioinformatics, nucleotide sequencesm, polymerase chain reaction

## Abstract

**Background and Aim:**

Infectious coryza (IC) or snot, is caused by *Avibacterium paragallinarum* and leads to upper respiratory disease in poultry. Various diagnostic methods are available, including isolation and identification through bacterial culture and biochemical tests. However, the isolation and subsequent identification of *A. paragallinarum* are challenging because the bacteria are fastidious and require specific growth factors. This study aimed to detect *A. paragallinarum* in clinical samples taken from the exudate of the infraorbital sinus of layer hens showing clinical signs of IC.

**Materials and Methods:**

Samples were collected from 10 layer hens with IC symptoms. Following DNA extraction, HPG-2 polymerase chain reaction (PCR) assays were performed. The PCR amplicons underwent electrophoresis to determine those of the correct target size (511 bp), and these were sequenced. The resultant sequences were analyzed using the National Center for Biotechnology Information (NCBI) basic local alignment search tool. MEGA X was used for bioinformatics analysis.

**Results:**

The presence of *A. paragallinarum* was confirmed by HPG-2 PCR in 4/10 samples. Bioinformatics analysis showed that the amino acid sequence of the samples and the *A. paragallinarum* reference sequences in the NCBI database were grouped within the same cluster. Furthermore, the nucleotide sequences showed 98.64%–100% of similarity with the reference sequences. The phylogenetic reconstruction of partial *pyr*G sequences from 55 *A. paragallinarum* strains/isolates deposited in GenBank confirmed that the four HPG-2 PCR-positive samples fell within the *A. paragallinarum* cluster, separate from the *Pasteurella multocida*, *Avibacterium* spp., and *Rodentibacter pneumotropicus* clusters.

**Conclusion:**

*Avibacterium paragallinarum* infection was molecularly confirmed in 4/10 (40%) samples by HPG-2 PCR amplicon detection. Clustering of the *pyr*G partial gene sequences revealed that the positive samples fell within the *A. paragallinarum* cluster.

## Introduction

*Avibacterium paragallinarum*, formerly known as *Haemophilus paragallinarum*, is the etiological agent of infectious coryza (IC) [1]. Due to taxonomical differences, the bacteria were designated as *A. paragallinarum* after investigating its phenotypes and genotypes [2]. This Gram-negative, polar-staining, and non-motile bacteria cause upper respiratory tract disease in chickens [3, 4]. Antibiotic sensitivity to *A. paragallinarum* varies from intermediate to sensitive [5]. The clinical signs of IC include facial swelling, purulent ocular and nasal discharge, swollen wattles, sneezing, dyspnea, decreased body weight, and inappetence [6]. However, these signs vary based on age and breed, and their duration and severity can be affected by external factors, such as poor housing management, the presence of parasites, malnutrition, and the presence of secondary infectious diseases, such as fowlpox, infectious bronchitis, infectious laryngotracheitis, *Mycoplasma gallisepticum*, and pasteurellosis [2].

Infectious coryza has a significant economic impact because it can lead to a decrease in egg production of up to 40% as well as increased culling of growing chickens [7, 8]. Various diagnostic methods have been developed for IC detection, including isolation and identification through bacterial culture and biochemical tests. However, culture is relatively difficult because the bacteria are fastidious and require specific growth factors. Many factors can cause culture failure, including the quality and type of samples, shipping conditions, and media type. Poor-quality media leads to a higher rate of culture failure, making polymerase chain reaction (PCR) a reliable diagnostic choice [9].

This study aimed to detect *A. paragallinarum* in clinical samples collected from the exudate of the infraorbital sinus of layer hens in the Special Region of Yogyakarta, showing clinical signs of IC Indonesia.

## Materials and Methods

### Ethical approval

The study was approved by Ethics Committee of Veterinary Medicine, Universitas Gadjah Mada (No: 080/EC-FKH/Int./2022). The samples were collected in accordance with standard collection procedures without hurting or necrotizing animals.

### Study period and location

The study was conducted from August 2022 to January 2023 in the Microbiology Department, Faculty of Veterinary Medicine, Universitas Gadjah Mada. The sequencing process was conducted at the integrated research and testing laboratory, Universitas Gadjah Mada.

### Sample collection and DNA extraction

Samples were collected from 10 layer hens from several farms in Sleman District, Special Region of Yogyakarta, Indonesia. All 10 layers showed signs of IC, including malodorous nasal discharge and facial edema. The exudate was swabbed and each sample was placed in a collection tube.

DNA was extracted from overnight cultures using a Presto Mini gDNA Bacteria Kit (Geneaid Biotech Ltd., Taipei, Taiwan). The isolates were transferred to a 1.5 mL microcentrifuge tube, centrifuged for 1 min at 14,000–16,000× *g*, and the supernatant was discarded. Then, guanidinium thiocyanate (GT) buffer (180 μL) was added, and the cell pellet was resuspended by vortex or pipette. Next, proteinase K (20 μL) was added, and the sample was incubated at 60°C for at least 10 min, during which the tubes were inverted every 3 min. Following incubation, GT buffer (200 μL) was added and mixed by vortex for 10 s, and then the sample was incubated at 70°C for at least 10 min to ensure the lysate was clear. At this time, the elution buffer (200 μL per sample) was preheated to 70°C. Next, 200 μL of absolute ethanol was added to the sample lysate and mixed immediately by vigorous shaking. The mixture was transferred to a genomic DNA (GD) column placed in a 2 mL collection tube and then centrifuged at 14,000–16,000× *g* for 2 min to bind the DNA and separate it from the supernatant. After discarding the flow-through collection tube, the GD column was placed in a new 2 mL collection tube. W1 buffer (400 μL) was added to the GD column, centrifuged at 14,000–16,000× *g* for 30 s, and discarded the flow-through. The GD column was reinserted into the 2 mL collection tube, and wash buffer (600 μL) was added. Following centrifugation at 14,000–16,000× *g* for 30 s, the flow-through was discarded. The GD column was placed back into the 2 mL collection tube and centrifuged for 3 min at 14,000–16,000× g. The dried GD column was then placed into a clean 1.5 mL microcentrifuge tube. To elute the pure DNA, the preheated elution buffer (100 μL) was added into the center of the column matrix, left for at least 3 min to allow thorough absorption, and then centrifuged at 14,000–16,000× *g* for 30 s.

### HPG-2 PCR

HPG-2 PCR, which targets a species-specific fragment of *A. paragallinarum* (~500 bp), was performed as described by Chen *et al*. [10] and Fauziah *et al*. [11] with some modifications to the PCR temperature. The primers used were F 5’- TGAGGGTAGTCTTGCACGCGAAT-3’ and R 3’-CAAGGTATCGATCGTCTCTCTACT-5’. Each PCR assay was performed in a final reaction volume of 50 μL containing 25 μL MyTaq HS Red Mix (Meridian Bioscience, OH, USA), 1.1 μL each of the forward and reverse primers, 5 μL of DNA template, and 17.8 μL ddH_2_O. The PCR protocol consisted of initial denaturation at 95°C for 15 min; followed by 30 cycles at 95°C for 60 s, 61°C for 60 s, and 72°C for 60 s; and a final extension at 72°C for 5 min and was performed using a BioRAD thermal cycler (USA). The PCR products were checked following 1.2% of agarose gel electrophoresis with FloroSafe staining in 100 mL of 1× Tris/Borate/EDTA buffer solution.

### Sequencing

The resultant PCR amplicons of the correct target size were purified and prepared for sequencing using a test tube. The final volume of each sequencing reaction was 20 μL. Each reaction comprised 1 μL template (10 ng), 1 μL of each primer (forward and reverse, 3.2 μm/μL), 2 μL Big Dye Terminator Ready Reaction Mix (Thermo fisher Scientific, USA), 12 μL nuclease-free water, and 5× sequencing buffer. Each reaction was mixed until evenly distributed and spun down. Cycle sequencing was carried out on a thermal cycler machine with initial denaturation at 96^o^C for 1 min, followed by 24–72 cycles of 96°C for 10 s, 50°C for 5 s, and 60°C for 4 min. Then, the samples were stored at 4°C before purification. Briefly, 10 μL of DNA from the cycle sequencing was added to 45 μL of SAM™ solution (Thermo Fisher Scientific) and 10 μL of BigDye X-Terminator (BDX-T was vortexed for 5 s beforehand) and mixed continuously for 30 min by vortexing. Then, the sample was centrifuged at 1000× *g* for 5 min. The supernatant (up to 20 μL) was removed, carefully and placed into wells, and the wells were closed with septa. Finally, the samples were centrifuged to remove air bubbles in the wells.

The sample plate was inserted into an ABI 3500 Genetic Analyzer (Applied Biosystems, USA) using the appropriate running plate settings (file name, polymer, 50 cm capillary, sequencing mode, and assay).

### Bioinformatic analysis

Scoring/base calling was performed using SeqA software (Applied Biosystems) with unstacked and ambiguous results of single nucleotide code sequences (ATGC) with multiple meanings or more nucleotide codes. The similarity of the determined sequences to those in GenBank was determined using the National Center for Biotechnology Information basic local alignment search tool (BLAST, NCBI) to search the BLASTN database. Bioinformatics analysis was performed using MEGA X software (https://www.megasoftware.net/downloads/dload_win_gui).

## Results

**Figure-1:**
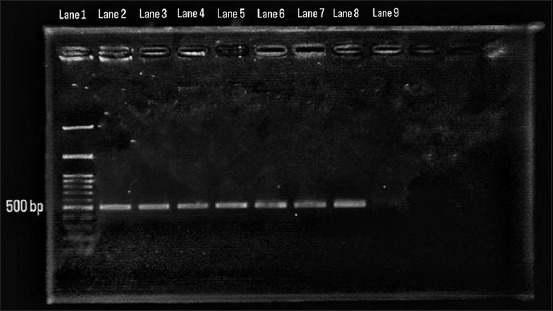
Lane 1 DNA ladder, Lane 2 control *Avibacterium paragallinarum* serotype A 221, Lane 3 Control *Avibacterium paragallinarum* serotype B SPROSS, Lane 4 control *Avibacterium paragallinarum* serotype C MODESTO, Lane 5 sample LP-9, Lane 6 LT-12, Lane 7 V-5, and Lane 8 LT -7, and Lane 9 negative control (primer without DNA).

In this study, samples were taken from 10 layer hens showing symptoms of IC. *Avibacterium paragallinarum* was detected by HPG-2 PCR (expected amplicon size, 511 bp) in 4/10 (40%) samples, namely, LP-9, LT-12, V-5, and LT-7 (Figure-1).

Basic local alignment search tool analysis of the obtained nucleotide sequences showed 98.64%–100% of similarity with the *A. paragallinarum* reference sequences submitted to GenBank, 88.11%–88.52% of similarity with *Avibacterium* spp., 88.02% of similarity with *Rodentibacter pneumotropicus*, and 83.94%–84.71% of similarity with *Pasteurella multocida*. Phylogenetic reconstruction revealed that the *A. paragallinarum* sequences detected in our samples fell into the same cluster. Comparison of nucleotide sequences from sample LP-9 with *A. paragallinarum* serotype C-Modesto (GenBank accession number DQ132874.1) reference sequence showed four single nucleotide polymorphisms (T-A at position 1373, G-T at position 1379, T-G at position 1381, and G-T at position 1382). The sequence of LT-12, LT-7, and V-5 was identical to the Modesto strain.

**Figure-2:**
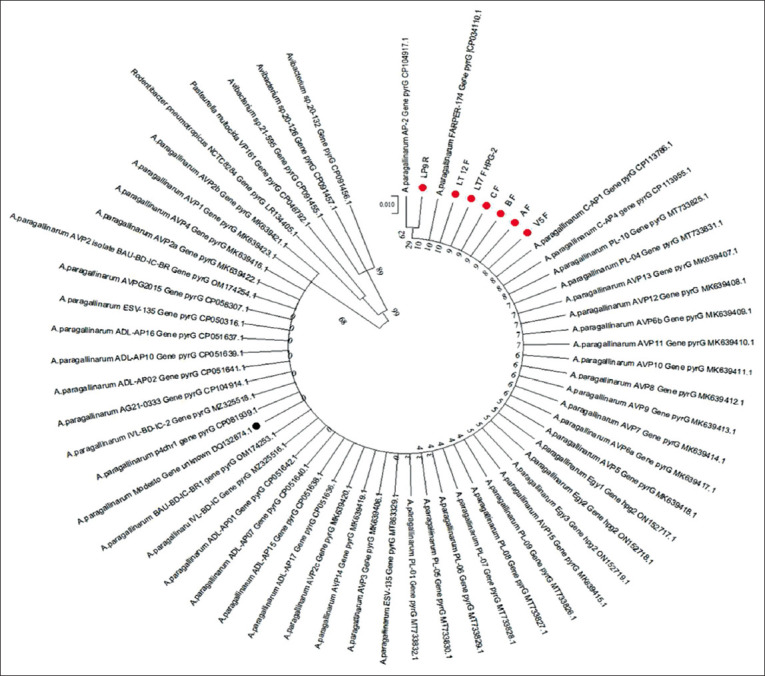
A phylogenetic tree reconstructed from partial sequences of *pyr*G nucleotide of *Avibacterium paragallinarum* with other strains submitted in national center for biotechnology information. The sample was marked with a red circle (AF, BF, and CF were reference isolates and LP9R, LT12F, LT-7 F HPG-2, and V-5 F were field isolates).

**Figure-3:**
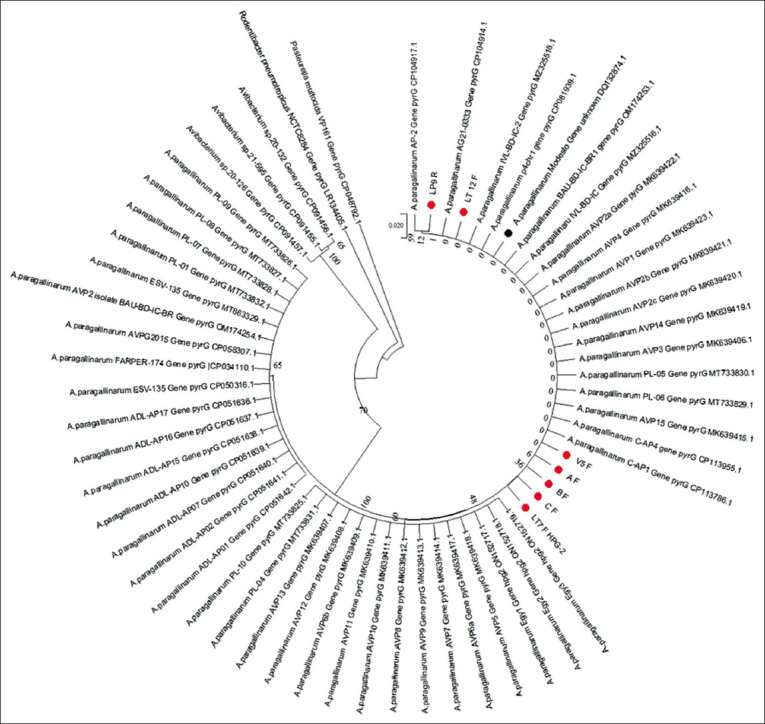
A phylogenetic tree reconstructed from partial sequences of *pyr*G amino acids of *Avibacterium paragallinarum* with other strains submitted in the national center for biotechnology information. The sample was marked with a red circle (AF, BF, and CF were reference isolates and LP9R, LT12F, LT-7 F HPG-2, and V-5 F were field isolates).

Phylogenetic reconstruction can be based on partial nucleotide or amino acid sequences. We phylogenetically analyzed partial nucleotide sequences (Figure-2) and partial amino acid sequences (Figure-3) of *pyr*G. Both phylogenetic reconstructions confirmed that our samples grouped with the cluster corresponding to *A. paragallinarum*, separate from the *P. multocida*, *Avibacterium* spp., and *R. pneumotropicus* clusters.

## Discussion

Infectious coryza is an important disease in poultry farming, particularly in countries with tropical or subtropical climates [12]. Thus, the identification and characterization of *A. paragallinarum* are essential for IC prevention and control [13]. Reports of field cases in Indonesia have shown that commercial vaccines cannot cover the incidence of IC [14]. Isolation and identification of *A. paragallinarum* remain relatively difficult because the bacteria are fastidious and slow growing. Furthermore, since it requires specific growth factors [15, 16], the cost of specialized media to culture and subsequently identify the bacteria is expensive [7]. Recently, the diagnostic options that can be used for IC have been expanded due to the development of species-specific PCR for the molecular identification of *A. paragallinarum* [17, 18]. During the present study, we collected samples from 10 layer hens suspected of IC from several farms that showed signs of upper respiratory tract infections, such as sneezing, nasal discharge, swollen face, and lacrimation. The most prominent symptoms of IC are acute to chronic inflammation of the upper respiratory tract, including the sinuses, with serous to mucoid nasal discharge, infraorbital sinus swelling, facial edema, conjunctivitis, and lacrimation [2, 19].

All 10 samples of infraorbital exudate were tested for IC using the species-specific HPG-2 PCR test for the molecular detection of *A. paragallinarum*. DNA extraction from direct swab preparations was used to increase the chance of detection. A swab sample of the mucus coming out of the nasal cavities was collected by gently squeezing the sinuses of the live birds [20]. An amplicon of the expected size (~500 bp) was detected in 4/10 samples (Figure-1). The HPG-2 PCR results were consistent with previous reports by Chen *et al*. [10], Anjaneya *et al*. [21], and Patil *et al*. [22]. Chen *et al*. [10] suggested that PCR is more sensitive than traditional bacterial isolation and identification methods. HPG-2 PCR successfully diagnosed *A. paragallinarum* infection after 60 days of storage at 20°C. In contrast, conventional culture methods could not detect isolates after 48 h of storage [21]. Polymerase chain reaction sensitivity is higher than traditional culture methods for the isolation and identification of *A. paragallinarum* [23]. Polymerase chain reaction detection in samples from layer hens was reported to have a positivity rate of 66.7% [24]. Using HPG-2 PCR tests can reduce the complexity and costs of the diagnostic process. Another advantage of HPG-2 PCR is its fast turnaround time, with results obtained within 24–48 h [25]. HPG-2 PCR amplicon sequence analysis showed a high similarity of up to 100% [12].

To construct a multiple sequence alignment for phylogenetic reconstruction, *pyr*G sequence data from 55 GenBank sequences of *A. paragallinarum* strains/isolates were edited, aligned, and analyzed in MEGA X. *pyr*G codes for cytidine triphosphate (CTP) synthase (glutamine hydrolyzing), which catalyzes the ATP-dependent amination of uridine triphosphate to CTP with either L-glutamine or ammonia as the nitrogen source. CTP synthase is involved in pyrimidine ribonucleotide/ribonucleoside metabolism. The enzyme catalyzes the following reaction: L-glutamine + H_2_O + UTP + ATP = CTP + phosphate + ADP + L-glutamate. The enzyme is a dimer of identical chains aggregating as a tetramer [26].

## Conclusion

HPG-2 PCR tests molecularly confirmed the presence of *A. paragallinarum* in 4/10 samples (40%). Phylogenetic reconstruction of partial sequences of *pyr*G revealed that all positive samples fell within the same cluster as the reference sequences.

## Authors’ Contributions

AETHW: Planned and designed the study. BS: Contributed to the design of research. FNP: Conducted the research, sampling, and analysis of the result, and prepared the manuscript under the guidance of AETHW and BS. All authors have read, reviewed, and approved the final manuscript.
